# CYP1B1 promotes tumorigenesis via altered expression of CDC20 and DAPK1 genes in renal cell carcinoma

**DOI:** 10.1186/s12885-015-1951-0

**Published:** 2015-12-01

**Authors:** Yozo Mitsui, Inik Chang, Shinichiro Fukuhara, Miho Hiraki, Naoko Arichi, Hiroaki Yasumoto, Hiroshi Hirata, Soichiro Yamamura, Varahram Shahryari, Guoren Deng, Darryn K. Wong, Shahana Majid, Hiroaki Shiina, Rajvir Dahiya, Yuichiro Tanaka

**Affiliations:** Department of Urology Shimane University Faculty of Medicine, 89-1 Enya-cho, 693-8501 Izumo, Japan; Department of Urology, San Francisco Veterans Affairs Medical Center and University of California San Francisco, Bldg 42 Rm 109, San Francisco, CA 94121 USA; Department of Oral Biology, Yonsei University College of Density, Seoul, South Korea; Department of Urology, Osaka University Graduate School of Medicine, 565-0871 Suita, Japan

**Keywords:** Cytochrome 450 1B1, Renal cell carcinoma, Apoptosis, CDC20, DAPK1

## Abstract

**Background:**

Cytochrome P450 1B1 (CYP1B1) has been shown to be up-regulated in many types of cancer including renal cell carcinoma (RCC). Several reports have shown that CYP1B1 can influence the regulation of tumor development; however, its role in RCC has not been well investigated. The aim of the present study was to determine the functional effects of *CYP1B1* gene on tumorigenesis in RCC.

**Methods:**

Expression of CYP1B1 was determined in RCC cell lines, and tissue microarrays of 96 RCC and 25 normal tissues. To determine the biological significance of CYP1B1 in RCC progression, we silenced the gene in Caki-1 and 769-P cells by RNA interference and performed various functional analyses.

**Results:**

First, we confirmed that CYP1B1 protein expression was significantly higher in RCC cell lines compared to normal kidney tissue. This trend was also observed in RCC samples (*p* < 0.01). Interestingly, CYP1B1 expression was associated with tumor grade and stage. Next, we silenced the gene in Caki-1 and 769-P cells by RNA interference and performed various functional analyses to determine the biological significance of CYP1B1 in RCC progression. Inhibition of CYP1B1 expression resulted in decreased cell proliferation, migration and invasion of RCC cells. In addition, reduction of CYP1B1 induced cellular apoptosis in Caki-1. We also found that these anti-tumor effects on RCC cells caused by CYP1B1 depletion may be due to alteration of CDC20 and DAPK1 expression based on gene microarray and confirmed by real-time PCR. Interestingly, CYP1B1 expression was associated with CDC20 and DAPK1 expression in clinical samples.

**Conclusions:**

CYP1B1 may promote RCC development by inducing CDC20 expression and inhibiting apoptosis through the down-regulation of DAPK1. Our results demonstrate that CYP1B1 can be a potential tumor biomarker and a target for anticancer therapy in RCC.

## Background

In 2014 kidney cancer affected nearly 63,920 people with an estimated 13,860 cancer related deaths in the United States [1]. Among the various forms of kidney cancers, renal cell carcinoma (RCC) is the most common, attributing about 85 % [2]. Recent advances in imaging technology and surgical techniques have contributed to the improvement of oncologic outcomes for patients with RCC. However, management of advanced RCC remains an extraordinary challenge because of limited therapeutic options and poor prognosis. Indeed, the 5-year survival rate for RCC patients with metastasis is less than 10 % [3]. In addition, about 30 % of localized RCC patients experience recurrence and/or metastases after curative radical surgery [4]. Thus, an increased understanding of the molecular basis of renal carcinogenesis may contribute to the development of better therapeutic and diagnostic strategies for the disease.

Cytochrome P450 1B1 (CYP1B1) belongs to the cytochrome P450 superfamily involved in the metabolism of a diverse range of endogenous and xenobiotic compounds including many carcinogens [5, 6]. CYP1B1 produces 4-hydroxy estrogens via hydroxylation of the parent estrogen [7], which has been postulated to play a major role in carcinogenesis by inducing DNA damage, mutation, and depurination [8]. Indeed, previous studies have demonstrated that CYP1B1 inhibition could prevent endometrial and head and neck carcinogenesis [9, 10]. Although CYP1B1 is expressed in normal tissues, it is expressed at much higher levels in many malignancies including RCC [11, 12]. In addition, elevated CYP1B1 enzymatic activity was found in RCC [13] despite the tumorigenicity of CYP1B1 in RCC has not been elucidated.

Dysregulated cellular proliferation is one of the most important factors that lead to tumorgenesis. In mitosis phase, the progression of the cell cycle is tightly regulated by the anaphase-promoting complex/cyclosome (APC/C), which is a downstream target of the mitotic spindle assembly checkpoint. The cell division cycle 20 homolog (CDC20), an essential regulator of cell division, complexes with APC/C which leads to the initiation of anaphase in early mitosis [14]. Therefore dysregulation of CDC20 may play important roles in cell growth and tumorigenesis. Recent studies have shown that CDC20 overexpression might be associated with an inappropriately functioning spindle assembly checkpoint, resulting in aneuploidy [15, 16]. Indeed, CDC20 overexpression has been linked to poor prognosis in lung [17], colon [18], bladder [19], gastric [20] and breast [21] carcinomas.

Apoptosis, a genetically controlled mechanism of cell death, is involved in embryonic development, tissue homeostasis and many diseases including cancer [22]. An imbalance between pro-apoptotic and anti-apoptotic factors induces an abnormal pattern of cell death. The death-associated protein kinase-1 (DAPK1), a serine/threonine kinase, is a p53 target gene and its activation may lead to apoptosis through activation of p53 [23, 24]. DAPK1 is widely expressed in normal tissues, while it is down-regulated in various malignancies [25–28].

Interestingly, a recent study has demonstrated that attenuation of *CYP1B1* expression has anti-proliferative and pro-apoptotic effects on endometrial cancer [9]. In light of this, we hypothesized that CYP1B1 may play a key role in renal carcinogenesis. The aim of this study was to identify the role of CYP1B1 in the pathogenesis of RCC. In this study, we confirmed that CYP1B1 expression was up-regulated in both RCC cells and samples. To validate the functional significance of *CYP1B1* overexpression, we depleted the gene in RCC cell lines by RNA interference and performed functional analysis. We also identified several key genes of the pathway involved in transformation and tumorigenesis based on gene microarray data. Finally, we found that *CDC20* and *DAPK1* are potentially regulated by *CYP1B1*.

## Methods

### Normal kidney sample and tissue microarray

Fresh frozen normal kidney tissue was purchased from BioServe (Beltsville, MD, USA). In total, 96 primary RCC comprised of 31 specimens from tissue microarray (TMA) KD951, 40 specimens from TMA KD485 (both acquired from US Biomax, Rockville, MD, USA), and 25 specimens from TMA CT565907 (ORIGENE, Rockville, MD, USA) were evaluated. Also, twenty-five normal kidney specimens were obtained from these 3 TMAs. Median patient age at surgery was 57 years old. Of the 96 RCC specimens, 83 were clear cell carcinoma and the remaining 13 specimens were non-clear cell carcinoma (3 papillary carcinoma, 3 chromophobe carcinoma, 3 sarcomatoid carcinoma, 2 granular carcinoma and 2 collecting duct carcinoma). Forty-seven (49.0 %) patients had Fuhrman grade 1 or 2, 15 (15.6 %) were grade 3 or 4, and the remaining 34 (35.4 %) were unknown. Forty (41.7 %) patients were stage I, 16 (16.7 %) patients stage II, 7 (7.3 %) stage III, 2 (2.1 %) stage IV, and the remaining 31 (32.2 %) were unknown.

### Cell lines and reagents

Renal cancer cell lines, Caki-1, Caki-2, A498, ACHN, 786-O and 769-P were obtained from the American Type Culture Collection (Manassas, VA, USA). McCoy’s 5A, MEM Eagle’s BSS (EMEM), RPMI 1640, Opti-MEM and penicillin/streptomycin mixture were obtained from the UCSF Cell Culture Facility (San Francisco, CA, USA). Fetal bovine serum (FBS) was a product of Atlanta Biologicals (Lawrenceville, GA, USA).

### Cell culture

Caki-1 and Caki-2 cells were cultured in McCoy’s 5A, and A498 and ACHN cells were maintained in EMEM medium while 786-O and 769-P cells were cultured in RPMI 1640 medium. All culture medium contained 10 % FBS and 100 μg/ml penicillin/streptomycin. All cell lines were maintained at 37 °C in a humidified atmosphere composed of 5 % CO_2_ and 95 % air.

### Knockdown of CYP1B1 in Caki-1 and 769-P cells

Oligonucleotides siRNA against human CYP1B1 and mismatch control oligonucleotides were purchased from Life technologies. For inhibition of CYP1B1, 5 μl of siRNA oligonucleotides (siRNA-CYP1B1 or siRNA-control) and 5 μl of lipofectamine RNAiMAX reagent (Invitrogen-Life Technologies Inc., Carlsbad, CA, USA) were separately diluted with 250 μl of Opti-MEM (Gibco, Carlsbad, CA, USA). Cells were then transfected with lipofectamine + siRNA-CYP1B1, lipofectamine + siRNA-control, or left untransfected. Transfection was terminated after 5 h by aspirating the transfection medium and adding fresh RPMI 1640 containing 10 % FBS. Non-adherent cells were washed off and the remaining cells were incubated at 37 °C.

### RNA extraction and quantitative RT-PCR

Total RNA was extracted from cultured cells using RNeasy Mini Kit (Qiagen) and was converted into cDNA by using an iScript^TM^ cDNA Synthesis Kit (BIO-RAD) according to the manufacturer’s instruction. To assess gene expression, cDNA was amplified with the TaqMan® Gene Expression Assays and TaqMan® Fast Universal PCR Master Mix using the 7500 Fast Real-Time PCR System. The target genes and their Assay ID were as follows: *CYP1B1* (HSpp16383_m1), *DKC1* (Hs00154737_m1), *OCLN* (Hs00170162_m1), *FASLG* (Hs00181225_m1), *MKI67* (Hs01032443_m1), *LPL* (Hs00173425_m1), *CA9* (Hs00154208_m1), *CDC20* (Hs00426680_mH), *TP73* (Hs00390315_m1), *BCL2A1* (Hs00187845_m1), *LTA* (Hs04188773_g1), *DAPK1* (Hs00234489_m1), *BIRC5* (Hs04194391_sH) and *GAPDH* (Hs03929097_g1). The relative level was calculated by the comparative *C*_t_ method (ΔΔ*C*_t_) using the 7500 Fast System Sequence Detection Software (Applied Biosystems).

### PCR assay of apoptosis and cancer pathways

cDNAs were evaluated for genes using the RT^2^ Profiler^TM^ PCR Array PAHS-012ZC (Human Apoptosis PCR Array) and the RT^2^ Profiler^TM^ PCR Array PAHS-033ZC (Human Cancer Pathway Finder^TM^ ) on the ABI Fast 7900 real-time PCR system with RT^2^ Real-time SYBR Green PCR master mix according to the manufacturer’s protocol.

### MTS assay

Cells were plated in triplicate in 96-well microplates at a density of 3 × 10^3^ cells per well. The number of viable cells was determined by adding 3-(4,5-dimethylthiazol-2-yl)-5-(3-carboxymethoxyphenyl)-2-(4-sulfophenyl)-2H-tetrazolium-based CellTiter 96 Aqueous One Solution Reagent (Promega, Madison, WI) to each well and measuring the absorbance at 490 nm on SPECTRA MAX 190 plate reader (Molecular Devices, Sunnyvale, CA, USA).

### Migration and invasion assay

Cell migration was evaluated by a wound-healing assay. Cells were plated in six-well dishes and the cell monolayers were scraped using a P-20 micropipette tip. Wound closure was monitored and the percent closure was measured. A cell invasion assay was carried out using modified Boyden Chambers consisting of transwell-precoated Matrigel membrane filter inserts with eight micro pores in 24-well tissue culture plates (BD Biosciences, Bedford, MA, USA). Cells were re-suspended in culture medium without FBS and placed in the upper chamber in triplicate. After 48 h incubation at 37 °C, cells migrating through the membrane were stained. The results were expressed as invaded cells quantified at OD 560 nm.

### Apoptosis assay

Fluorescence-activated cell-sorting (FACS) analysis for apoptosis was done 48 h post-transfection, using an annexin V-fluorescein isothiocyanate (FITC)/7-amino-actinomycin D (7-AAD) staining system obtained from BD Biosciences (San Diego, CA, USA) and a Cell Lab Quanta^TM^ SC MPL (Beckman Coulter, Fullerton, CA, USA). Cells were stained with annexin V-FITC only (early apoptotic) or both annexin V-FITC and 7-AAD (late apoptotic) and considered to be total apoptotic cell fractions.

### Western analyses

Normal kidney tissue and whole cell extracts were prepared using radioimmunoprecipitation assay buffer (RIPA; Thermo Scientific, Rockford, IL, USA) containing protease inhibitor cocktail (Roche Diagnostics, Basel, Switzerland). Protein quantification was done using a BCA protein assay kit (Pierce) according to the manufacturer’s instructions. Total cell protein (15–20 μg) was used for Western blotting. Samples were run on the polyacrylamide gels and then transferred to PVDF membranes. The membranes were immersed in 3 % skim milk in antibody against CYP1B1 (#ab32649, Abcam, Cambridge, MA, USA), CDC20 (#4823, Cell Signaling Technology), DAPK1 (#3008, Cell Signaling Technology) and GAPDH (#sc-32233, Santa Cruz) overnight at 4 °C. Blots were washed in TBS containing 0.1 % Tween20 and labeled with horseradish peroxidase conjugated secondary anti-rabbit antibody (Cell Signaling Technology). Specific complexes were visualized with an enhanced chemiluminescence (ECL) detection system (GE Healthcare, Little Chalfont, UK) using the Chemidoc imaging system (Bio Rad, CA, USA). Protein expression levels were expressed relative to GAPDH.

### Immunohistochemical analyses

Immunostaining of CYP1B1, CDC20 and DAPK1 were performed on TMA slides using UltraVision Detection System (Thermo Scientific) according to the manufacture’s instruction, and 2 TMA slides were evaluated for each antibody. After 12 h incubation with rabbit polyclonal antibody for CYP1B1 (1:1500, #ab32649, Abcam), CDC20 (1:100, #ab86104, Abcam) and DAPK1 (1:250, #ab109382, Abcam), 3, 3'-diaminobenzidine (DAB) was added as chromogen followed by counterstaining with hematoxylin. The degree of immunostaining was evaluated by two independent observers who were blind to the clinical data of the TMAs. For CYP1B1, cytoplasmic expression was analyzed by the intensity of positive cells using Image J software (http://rsb.info.nih.gov/ij) and was ranked on an overall scale from 0 to 3; with 0 indicating the absence of staining; 1, weak staining; 2, moderate staining; and 3, strong staining [12]. CDC20 and DAPK were scored by multiplication of the percentage of positively stained tumor cells and staining intensity according to previous studies [17, 18, 28]. Briefly, the percentage of positive cells was scored as: 0, 0 %; 1+, 1–10 %; 2+, 11–50 %; or 3+, 51–100 %. The intensity of nuclear staining and/or cytoplasmic staining was scored as follows: grade 0, negative; 1+, weakly positive; 2+, moderately positive; or 3+, strongly positive. The two scores were then multiplied to calculate the final score (range from 0 to 9).

### Statistical analysis

Values are presented as the mean ± standard error mean based on results obtained from at least three independent experiments. All data were analyzed by the StatView version 5 statistical software (SAS Institute, Inc., Cary, NC). The relationship between two variables and the numerical values were analyzed using the two-tailed unpaired Student’s *t*-test. Chi-square test was used for analyzing the correlation between clinicopathologic parameters and CYP1B1 expression. A *p*-value of less than 0.05 was considered to be statistically significant.

### Ethics statement

All samples obtained with informed consent according to US federal law were purchased commercially and at the laboratory in San Francisco, specimens and de-identified patient data were used for analysis. This study was approved by the Clinical Research Office of the San Francisco Veterans Affairs Medical Center and the Institutional Review Board (study number 10–03240) of the University of California at San Francisco.

## Results

### CYP1B1 is up-regulated in RCC cell lines and RCC tissues

**Fig. 1:**
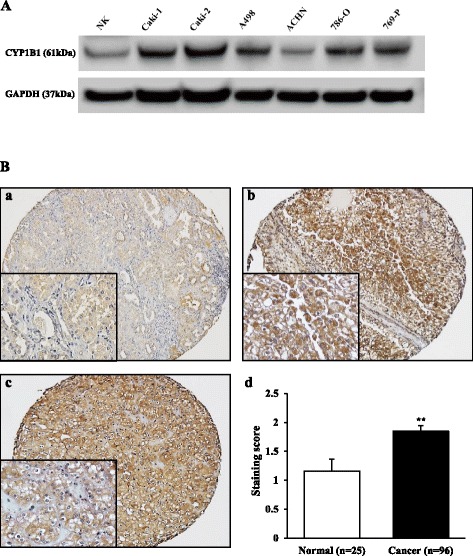
CYP1B1 expression in RCC cell lines and tissues. **a** Representative immunoblot displaying CYP1B1 expression in normal kidney (NK) sample, Caki-1, Caki-2, A498, ACHN, 786-O and 769-P. CYP1B1 protein was up-regulated in RCC cell lines in comparison with that of NK. **b** Representative immunostaining of CYP1B1 in clinical sample obtained from tissue microarray. (*a*) Weak cytoplastic staining of CYP1B1 was observed in the normal renal tubules. (*b*) Strong cytoplasmic staining of CYP1B1 was observed in clear cell RCC tumors. (*c*) CYP1B1 overexpression was also observed in chromophobe RCC tumors. (*d*) CYP1B1 staining score in clinical samples. CYP1B1 protein expression in RCC samples was significantly higher than that of normal kidney tissues . **, *P* < 0.01

To determine CYP1B1 protein expression, Western analysis was performed using normal kidney tissue, Caki-1, Caki-2, A498, ACHN, 786-O, and 769-P cells. CYP1B1 protein expression was significantly up-regulated in most RCC cell lines compared with normal kidney (Fig. 1a). Next, RCC TMA consisting of 96 cases of primary RCC was immunostained with CYP1B1 antibody. While CYP1B1 expression was weak or not detected in most of the normal kidney tissues, the majority of RCC samples showed moderate or strong CYP1B1 immunoreactivity with an average staining score of 1.84 ± 0.10 (versus 1.16 ± 0.21 in normal kidney tissues) (*p* < 0.01; Fig. 1b).

The clinicopathological status of the 96 RCC samples is shown in Table 1. RCC were classified into 2 groups according to CYP1B1 staining score; High (2–3) and Low (0–1). Increased expression of CYP1B1 was correlated with high grade or high stage disease, with a trend toward statistical significance (*P* = 0.0875, *P* = 0.0692, respectively). However, there were no significant differences for age, gender, histological type, lymph node and systematic metastasis between the groups.

**Table 1:** CYP1B1 expression in relation to clinicopathological findings

	CYP1B1 staining score			
Variables	Low (*n* = 34)		High (*n* = 62)	*P* value
Median age (yrs)	58		57	0.2264
Gender				
male	24 (70.6 %)		42 (67.7 %)	0.7735
female	10 (29.4 %)		20 (32.3 %)	
Histology				
clear cell	30 (88.2 %)		53 (85.5 %)	0.7063
non-clear cell	4 (11.8 %)		9 (14.5 %)	
Fuhrman grade				0.0875
1–2	21 (61.7 %)		26 (41.9 %)	
3–4	3 (8.9 %)		12 (19.4 %)	
unknown	10 (29.4 %)		24 (38.7 %)	
Stage				0.0692
I–II	24 (70.6 %)		32 (51.6 %)	
III–IV	1 (2.9 %)		8 (12.9 %)	
unknown	9 (26.5 %)		22 (35.5 %)	
Lymph node metastasis				0.5085
negative	23 (67.7 %)		32 (51.6 %)	
positive	1 (2.9 %)		3 (4.8 %)	
unknown	10 (29.4 %)		27 (43.6 %)	
Systematic metastasis				0.2180
negative	25 (73.5 %)		37 (59.7 %)	
positive	2 (5.9 %)		8 (12.9 %)	
unknown	6 (17.6 %)		17 (27.4 %)	

### Attenuation of *CYP1B1* expression inhibits renal cancer cell viability, migration, and invasion

**Fig. 2:**
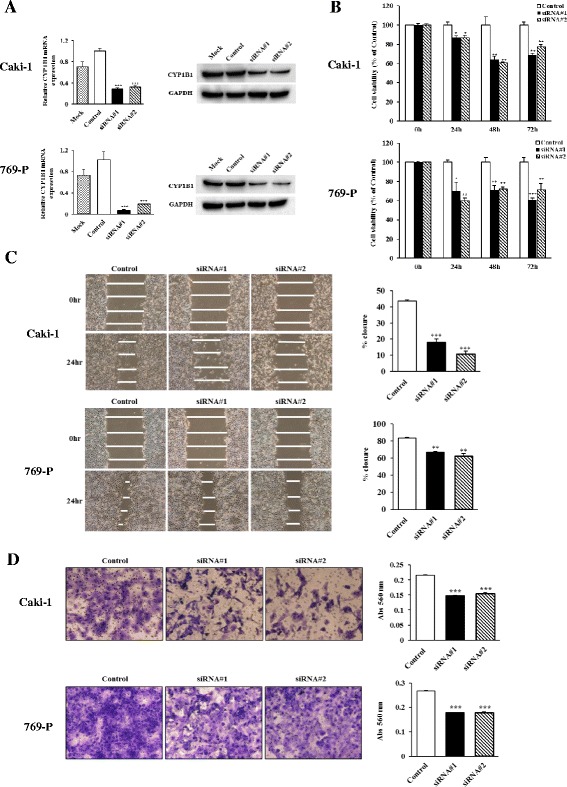
Effect of CYP1B1 knockdown on cell proliferation, migration and invasion in RCC cell lines. **a** Knockdown of CYP1B1 levels in RCC cell lines (Caki-1 and 769-P) were determined by real time RT-PCR and Western immunoblot analysis at 48 h after transfection with two different *CYP1B1* siRNAs. **b** Cell viability was analyzed by the MTS cell proliferation assay 0, 24, 48 and 72 h after siRNA treatment. Attenuation of CYP1B1 significantly inhibited cell viability in both cell lines. *, *P* < 0.05. **, *P* < 0.01.***, *P* < 0.001. **c** Representative images of wound healing assay. After siRNA transfection for 48 h, a wound was formed by scraping and closure of wound measured after 24 h. Attenuation of CYP1B1 significantly inhibited cell migration. **, *P* < 0.01.***, *P* < 0.001. **d** Representative images of invasion assay. Down-regulation of CY1B1 significantly decreases cell invasion. ***, *P* < 0.001

CYP1B1 levels were increased in RCC and thus, the functional significance of this gene were explored. This was done by examining whether reduction of CYP1B1 expression has an effect on cell viability, migration, or invasion properties of RCC cell lines. After transfection with two different *CYP1B1* siRNAs, significant reduction of CYP1B1 mRNA and proteins were detected in both Caki-1 and 769-P cells (Fig. 2a). Cell proliferation (Fig. 2b) and wound healing assays (Fig. 2c) demonstrated significant inhibition in CYP1B1 transfectants in both Caki-1 and 769-P cells compared to the control siRNA transfectants. Matrigel invasion assay also showed that the number of invaded cells was significantly decreased in CYP1B1 transfectants compared with their control counterparts after 24 h (Fig. 2d). These results suggest that CYP1B1 plays an important role in RCC progression.

### CYP1B1 influences cellular apoptosis in RCC cells

**Fig. 3:**
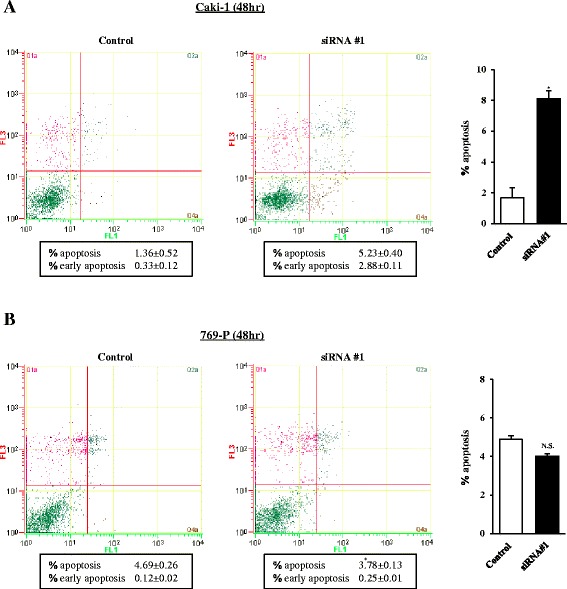
Effect of CYP1B1 knockdown on apoptosis. Apoptosis assays with Caki-1 (**a**) and 769-P (**b**) cells were done 48 h post siRNA transfection. Representative biparametric histogram showing cell population in early (*bottom right quadrant*) and late (*top right quadrant*) apoptotic and viable (*bottom left quadrant*) states. The bar chart indicates the ratio of apoptotic cell fractions (early plus apoptotic cells) in CYP1B1 transfectants compared with controls. Apoptotic cell fractions are expressed as relative value composed to the average expression of control siRNA transfectant. *, *P* < 0.05

Since attenuation of *CYP1B1* expression significantly inhibited cell growth and progression of RCC cells, we hypothesized that its expression may induce apoptosis. Apoptosis was examined in control siRNA-treated cells or *CYP1B1* siRNA-treated cells. Results of apoptosis assay in Caki-1 and 769-P cells done 48 h post-transfection are shown in Fig. 3. In Caki-1 cells, the apoptotic and early apoptotic fractions (upper right and lower right in the quadrant images, respectively) were significantly greater in *CYP1B1* -depleted cells (2.88 % + 5.23 %) compared to control cells (0.33 % + 1.36 %) (*p* < 0.05; Fig. 3a). In contrast, these differences were not seen in 769-P cells (Fig. 3b).

### CYP1B1 regulates the expression of *CDC20* and *DAPK1* in RCC cell lines

**Fig. 4:**
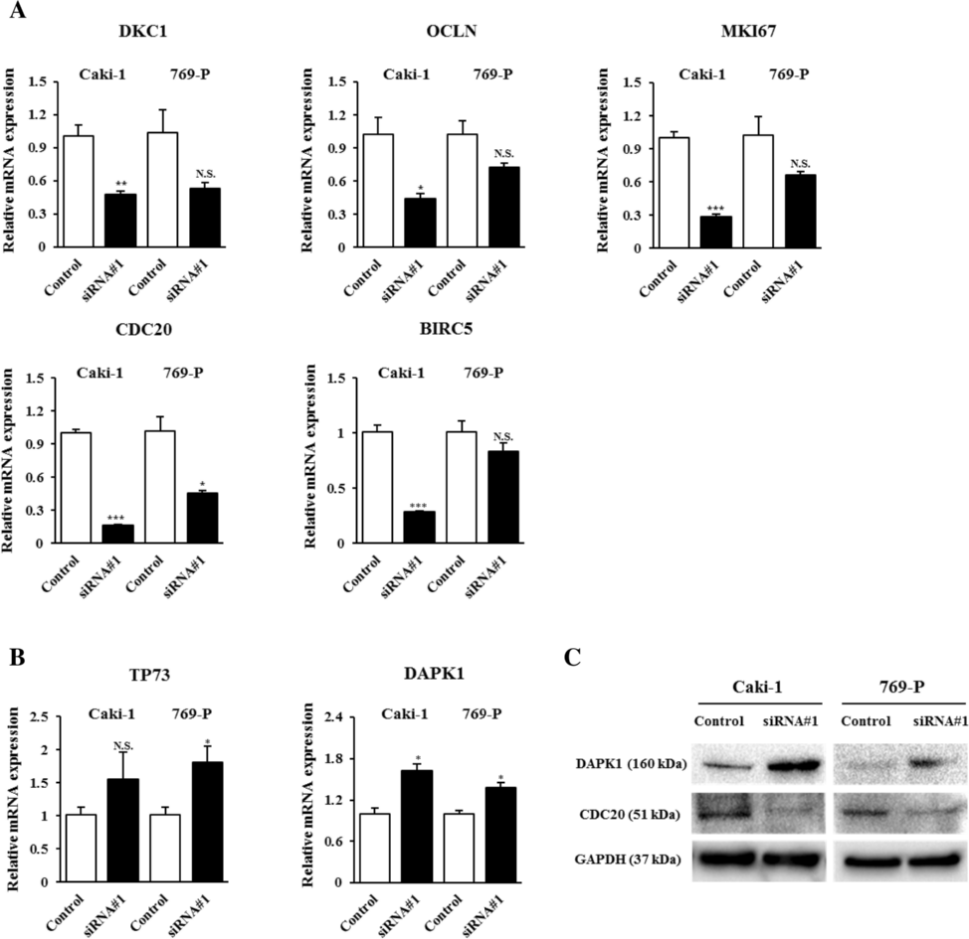
Verification of cDNA microarray data by RT-PCR and Western blotting. **a** Among 9 down-regulated genes, 6 genes (*DKC1*, *OCLN*, *MIK67*, *LIG4*, *CDC20*, *BIRC5*) were confirmed by real-time PCR using Taqman probe in Caki-1 cells. Of these 6 genes, *CDC20* was down-regulated in both Caki-1 and 769-P cells. *, *P* < 0.05. **, *P* < 0.01.***, *P* < 0.001. **b** Among 4 up-regulated genes, *TP73* and *DAPK1* were confirmed by real-time PCR and only *DAPK1* was down-regulated in both cell lines. *, *P* < 0.05. (C). Western immunoblotting analysis of DAPK1, LIG4 and CDC20 in control and CYP1B1 transfected Caki-1 and 769-P cells. A positive correlation was found between protein levels and mRNA expression of DAPK1 and CDC20 in both cell lines. GAPDH was used as a loading control

To further understand the precise mechanism of the anti-tumor effect on RCC cells induced by *CYP1B1* knockdown, we looked for changes in gene expression in *CYP1B1* siRNA-treated and *NS* siRNA-treated control cells using the Human Cancer Pathway RT^2^ Profiler^TM^ PCR Array and the Human Apoptosis RT^2^ Profiler^TM^ PCR Array. From the cancer pathway-related genes analyzed, eight were down-regulated ~ 2- to 5- fold after *CYP1B1* depletion and among them, *CDC20* was the greatest (Table 2). To verify array data, we performed real-time PCR using Taqman probes. Consistently, a robust decrease of *CDC20* mRNA expression was detected in *CYP1B1* siRNA-treated Caki-1 (6.1- fold) and 769-P (2.2- fold) (Fig. 4a). However, a significant decrease of *DKC1* (Dyskeratosis congenita1), *OCLN* (Occuludin), and *MIK67* (Antigen identified by monoclonal antibody Ki-67) expression were seen in only Caki-1 cells (Fig. 4a). We also examined the protein expression of CDC20 to identify the association with their mRNA expression levels. As shown by Western blotting, CDC20 protein levels were significantly decreased in *CYP1B1* siRNA-treated cells compared with *NS* siRNA-treated control cells (Fig. 4c). These results indicate that *CDC20* is potentially regulated by *CYP1B1* in RCC.

**Table 2:** Cancer pathway-related and apoptosis-related genes significantly altered in Caki-1

Refseq	Symbol	Description	Fold Change
Cancer pathway-related genes			
NM_005994	DKC1	Dyskeratosis congenita 1, dyskerin	0.4918
NM_002982	OCLN	Occludin	0.4816
NM_001147	FASLG	Fas ligand (TNF superfamily, member 6)	0.4107
NM_005983	MKI67	Antigen identified by monoclonal antibody Ki-67	0.3950
NM_001315	LPL	Lipoprotein lipase	0.3660
NM_001950	LIG4	Ligase IV, DNA, ATP-dependent	0.3572
NM_018975	CA9	Carbonic anhydrase IX	0.2484
NM_001002	CDC20	Cell division cycle 20 homolog (S. cerevisiae)	0.1879
Apoptosis-related genes			
NM_005427	TP73	Tumor protein p73	4.3079
NM_004049	BCL2A1	BCL2-related protein A1	2.2191
NM_000595	LTA	Lymphotoxin alpha (TNF superfamily, member 1)	2.1273
NM_004938	DAPK1	Death-associated protein kinase 1	2.0505
NM_016252	BIRC6	Baculoviral IAP repeat containing 6	0.5000
NM_003805	CRADD	CASP2 and RIPK1 domain containing adaptor with death domain	0.4386
NM_003842	TNFRSF10B	Tumor necrosis factor receptor superfamily, member 10b	0.4368
NM_003844	TNFRSF10A	Tumor necrosis factor receptor superfamily, member 10a	0.4087
NM_001924	GADD45A	Growth arrest and DNA-damage-inducible, alpha	0.3821
NM_014430	CIDEB	Cell death-inducing DFFA-like effector b	0.2975
NM_001066	TNFRSF1B	Tumor necrosis factor receptor superfamily, member 1B	0.2862
NM_003806	HRK	Harakiri, BCL2 interacting protein (contains only BH3 domain)	0.2360
NM_000594	TNF	Tumor necrosis factor	0.2126
NM_001561	TNFRSF9	Tumor necrosis factor receptor superfamily, member 9	0.1852
NM_001168	BIRC5	Baculoviral IAP repeat containing 5	0.1369

Table 2 displays genes that were up-regulated and down-regulated 2 or greater due to *CYP1B1* knockdown in apoptosis-related gene array analyses. Among 4 up-regulated genes, only *DAPK1* was found to be significantly increased in both Caki-1 (1.6- fold) and 769-P (1.3- fold) cells by real-time PCR (Fig. 4b). As shown in Fig. 4c, an increase in DAPK1 protein was also found in both *CYP1B1* siRNA-treated RCC cells. Though *TP73* (Tumor protein p73) was observed to be most increased by array analyses, its up-regulation was not confirmed in Caki-1 by real-time PCR (Fig. 4b). Among 15 down-regulated genes, *BIRC5* (Baculoviral IAP repeat containing 5) was observed to be the lowest. However, a significant decrease of *BIRC5* mRNA expression was detected only in *CYP1B1* siRNA-treated Caki-1 (Fig. 4b). Thus, these data indicate that the attenuation of *CYP1B1* expression caused induction of *DAPK1* and could lead to apoptosis in RCC.

### CYP1B1 expression is associated with CDC20 and DAPK1 expression in clinical samples

**Fig. 5:**
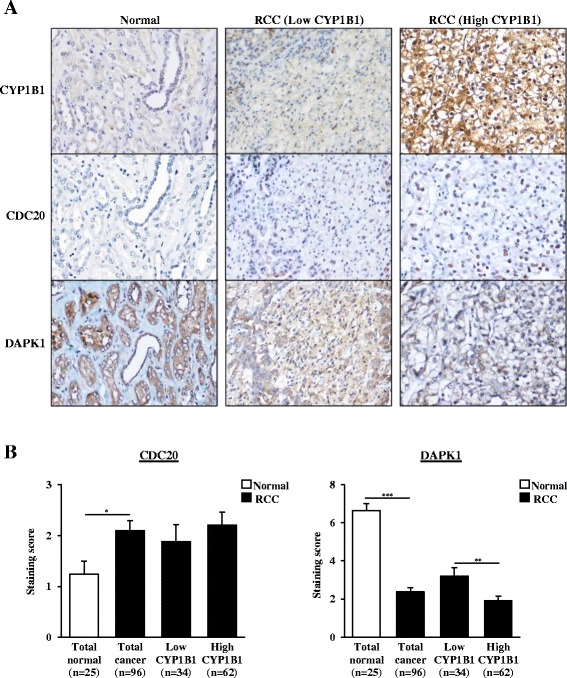
Expression of CDC20 and DAPK1 and their correlation with CYP1B1 in clinical samples. **a** Representative immunostaining of CDC20, DAPK1 and CYP1B1 from matched samples including normal tissue, Low CYP1B1 RCC and High RCC tissues. Intracellular CDC20 accumulation was positive in some cancer cells, whereas majority of the normal kidney cells were negative. Strong cytoplasmic staining of DAPK1 was more common in the normal kidney tissues than in RCC samples. **b** CDC20 expression was significantly higher in RCC than normal kidney tissues. There was a positive correlation between the expression of CDC20 and CYP1B1 in RCC, though it did not reach statistical significance. DAPK1 expression was significantly down-regulated in RCC as compared to normal tissues. The expression level of DAPK1 was also significantly correlated with Low and High CYP1B1 expression in RCC. *, *P* < 0.05. **, *P* < 0.01.***, *P* < 0.001

To validate the correlation between CYP1B1 expression and CDC20 or DAPK1 expression, we performed immunohistochemical studies using clinical samples. Case specimens were grouped as Low or High CYP1B1 expression categories as previously determined (Table 1). Representative CDC20 and DAPK1 immunostaining pattern are shown in Fig. 5a. RCC samples showed a higher level of CDC20 expression in comparison with normal kidney specimens (Fig. 5b). In addition, the High CYP1B1 group in RCC showed higher CDC20 expression than Low CYP1B1 group, though the difference did not reach statistical significance (Fig. 5a, b). As for DAPK1, strong cytoplasmic staining was more common in the normal kidney samples than in the RCC tissues (Fig. 5a, b). Moreover, RCC samples with Low CYP1B1 expression had a significantly higher level of DAPK1 expression as compared to those with High CYP1B1 expression (*p* < 0.01; Fig. 5a, b).

## Discussion

It is recognized that CYP1B1 is up-regulated and plays an essential role in carcinogenesis in several types of cancers [9–11]. CYP1B1 catalyzes the hydroxylation of estrogens to 4-hydroxy-estrogens as well as convert polycyclic aromatic hydrocarbons to mutagenic forms that play a causative role in the carcinogenesis process [8, 29]. When 4-hydroxy-estradiol was injected into animals, tumors were formed in the kidneys [30, 31]. Therefore, it is not surprising that CYP1B1 is actively involved in RCC. Here, we investigated the effects of CYP1B1 in renal carcinogenesis by performing functional assays and gene microarray analyses.

CYP1B1 is expressed at high levels and its enzyme activity is significantly elevated in RCC [11–13]. In addition, its overexpression contributes to the decreased sensitivity to anticancer drugs, such as paclitaxel and docetaxel [32, 33]. Recently, we demonstrated that CYP1B1 up-regulation could induce docetaxel resistance in RCC cells [12]. In this report, we confirmed that CYP1B1 protein expression was up-regulated in most RCC cell lines. Likewise, levels of immunoreactive protein of CYP1B1 were found to be significantly higher in clinical RCC tissues than normal kidney tissues. Next, we examined the association between CYP1B1 expression and clinicopathological findings in RCC samples. Importantly, CYP1B1 over-expression was correlated with high grade and advanced stage, though it did not reach statistical significance. Thus, CYP1B1 is demonstrated to have a potential prognostic and diagnostic value in RCC.

Attenuation of *CYP1B1* expression has been found to decrease the proliferative activity, cell migration capability and invasiveness of endometrial and head and neck cancer cell lines [9, 10]. In our present study, *CYP1B1* depletion also strongly inhibited these activities in RCC cell lines. Aberration on the cell cycle is a well-known mechanism underlying uncontrolled cellular proliferation, which leads to tumor formation. The spindle assembly checkpoint plays important roles in mitosis by preventing chromosome missegregation [34]. CDC20, known as a key component of the spindle assembly checkpoint proteins, is shown to drive mitosis from metaphase to anaphase by activating a subunit of APC/C [14]. Previous studies have demonstrated that knockdown of CDC20 expression could lead to decreased cellular proliferation in tumor cells [35, 36], and its overexpression is associated with poor prognosis in many human cancers [17–21]. In our observation from immunohistochemistry, CDC20 was significantly up-regulated in RCC tissue in comparison with normal kidney tissues. In addition, we found that *CYP1B1* knockdown in RCC cell lines leads to altered expression of *CDC20*. Furthermore, there was a trend that CDC20 expression was at a higher level in the High CYP1B1 group as compared to the Low CYP1B1 group. Interestingly, the expression of Ki-67, an important proliferative biomarker reflecting oncologic outcomes including RCC [37, 38], was reported to be correlated with CDC20 expression [21]. We found that *MKI67* mRNA expression was also down-regulated in *CYP1B1* siRNA-treated RCC cells. Considering these findings, we believe that CDC20 is closely associated with RCC tumorigenesis and potentially regulated by CYP1B1.

Defects in apoptotic pathways promote tumor initiation, progression and metastasis [39]. Apoptosis is hence considered to be a major causative factor in tumorigenesis. In this study, we clearly demonstrated that down-regulation of CYP1B1 induced significantly increased levels of apoptosis in Caki-1 cells. Also, we sought to determine the genes associated with down-regulated CYP1B1-mediated apoptosis and observed overexpression of *DAPK1* after *CYP1B1* depletion in RCC cell lines based on gene microarray analyses. Several studies have shown that *DAPK1* is a tumor-suppressor gene and down-regulated in many types of cancers [25–28]. In addition, an *in vitro* experiment revealed that DAPK1 enzyme activity was reduced in RCC [40]. We found that levels of DAPK1 protein were significantly lower in RCC than normal tissue, and there was an inverse correlation between protein levels of CYP1B1 and DAPK1. These results indicate that *DAPK1* may be regulated by *CYP1B1* and the functional role of DAPK1 in RCC is that of a tumor suppressor.

DAPK1 leads to p53 activation and apoptosis through DAPK1 phosphorylation of tetrametric p53 on Ser20, which is located within the transactivation domain that binds p300 [41]. In addition, DAPK1 indirectly induces p53 activation by activating the ARF tumor suppressor, which inhibits MDM2, an inhibitor of p53 [23]. Thus, the tumor suppressive mechanism of DAPK1 is tightly involved in p53-dependent apoptosis. Also, it is well known that mutations in p53 lose wild-type p53 tumor suppressor activity and gain oncogenic ability. We hypothesize that this may explain why restoration of DAPK1 through CYP1B1 reduction did not induce an apoptotic effect in 769-P cells since they have a p53 mutation.

The expression of DAPK1 is thought to be down-regulated by DNA promoter methylation and associated with poor prognosis in a variety of tumors [25–28]. Promoter CpG hypermethylation appears to be a common mechanism underlying gene silencing and many tumor-related genes are also silenced by DNA hypermethylation in RCC [42, 43]. The correlation between methylation status of *DAPK1* and tumor progression in RCC has been demonstrated [44, 45], although other studies have shown that its expression may not be regulated by DNA promoter methylation [40]. Further research is needed to clarify the precise mechanism of *DAPK1* restoration through the depletion of *CYP1B1*.

## Conclusions

In summary, our study demonstrates that CYP1B1 depletion reduces tumorigenesis in RCC cell lines. In clinical RCC samples, CYP1B1 expression seemed to be associated with unfavorable prognostic factors. CYP1B1 may promote RCC development by inducing CDC20 expression and inhibiting apoptosis through the down-regulation of DAPK1. In view of these results, we conclude that CYP1B1 may be an attractive target for designing improved diagnostic and therapeutic strategies for RCC.

## Abbreviations

CDC20: the cell division cycle 20 homolog
CYP1B1: cytochrome P450 1B1
DAPK1: the death-associated protein kinase-1
RCC: renal cell carcinoma
TMA: tissue microarray.
