# Correction: CYP1B1 promotes tumorigenesis via altered expression of CDC20 and DAPK1 genes in renal cell carcinoma

**DOI:** 10.1186/s12885-022-09907-4

**Published:** 2022-07-25

**Authors:** Yozo Mitsui, Inik Chang, Shinichiro Fukuhara, Miho Hiraki, Naoko Arichi, Hiroaki Yasumoto, Hiroshi Hirata, Soichiro Yamamura, Varahram Shahryari, Guoren Deng, Darryn K. Wong, Shahana Majid, Hiroaki Shiina, Rajvir Dahiya, Yuichiro Tanaka

**Affiliations:** 1 Department of Urology Shimane University Faculty of Medicine, 89-1 Enya-cho, Izumo, 693-8501 Japan; 2 Department of Urology, San Francisco Veterans Affairs Medical Center and University of California San Francisco, Bldg 42 Rm 109, San Francisco, CA 94121 USA; 3 Department of Oral Biology, Yonsei University College of Density, Seoul, South Korea; 4 Department of Urology, Osaka University Graduate School of Medicine, Suita, 565-0871 Japan

**Correction: BMC Cancer 15, 942 (2015)**

**https://doi.org/10.1186/s12885-015-1951-0**

Following publication of the original article [1], the authors identified an error in Fig. 2C.

**Fig. 2:**
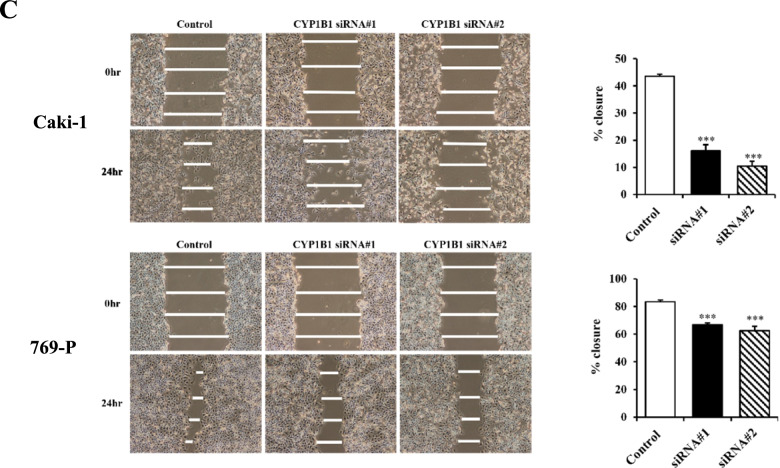
c Representative images of wound healing assay. After siRNA transfection for 48 h, a wound was formed by scraping and closure of wound measured after 24 h. Attenuation of CYP1B1 significantly inhibited cell migration. **, *P* < 0.01.***, *P* < 0.001

In Fig. 2C, the wrong image representing wound healing in CYP1B1-siRNA#1-treated Caki-1 cells at 0 hours was inadvertently applied. Although the wrong image was mistakenly utilized, results are essentially the same and does not change the outcome or conclusions of the article. The correct Fig. 2C is given in this correction article.
